# Respiratory Outcome of the Former Premature Infants

**DOI:** 10.25122/jml-2019-0123

**Published:** 2019

**Authors:** Raluca Daniela Bogdan, Lidia Rusu, Adrian Ioan Toma, Leonard Nastase

**Affiliations:** 1. Medicover Hospital, Bucharest, Romania; 2. Regional Center of Public Health, Iasi, Romania; 3. Life Memorial Hospital , Bucharest, Romania; 4. Alessandrescu - Rusescu National Institute of Mother and Child Health, Bucharest, Romania

**Keywords:** Premature infants, recurrent wheezing, RSV infection, viral infections, respiratory outcome

## Abstract

The research aims to identify the respiratory pathology during the first two years of life in premature infants with gestational ages between 30-34 weeks and the risk factors for these conditions (familial, prenatal, and neonatal).

There were investigated 31 premature infants with gestational ages between 30-34 weeks and the incidence of bronchopulmonary dysplasia, infections with the respiratory syncytial virus, or other viral infections requiring hospitalization, recurrent wheezing, and nasal colonization with pathogenic bacteria were noted. Also, regression models for each type of respiratory pathology as a function of the antenatal (smoking in the family, atopy, mother’s age) and neonatal (gestational age, respiratory distress syndrome, duration of the treatment with antibiotics, use of the reserve antibiotics) factors were elaborated.

Respiratory distress syndrome was present in 20 premature infants, and 19 infants received respiratory support. Two former premature infants presented with bronchopulmonary dysplasia, 3 with severe respiratory syncytial virus infections, 7 with recurrent wheezing, and 16 with viral infections requiring hospitalization. Respiratory distress syndrome and severe viral infections were more frequently found in families of smokers. Low gestational age and familial atopy were identified as good predictors of severe respiratory syncytial virus infections (p< 0.03)

Premature infants with gestational ages between 30-34 weeks present with the risk of appearance of respiratory diseases during the first two years of life, especially disorders of the airways. Familial atopy and low gestational age represent independent risk factors for severe respiratory syncytial virus infections.

## Introduction

The long-time development of the premature infant is regarded most frequently from the perspective of the neurologic prognosis [1]. However, there are more and more studies that show that the former premature infants present significant risks regarding respiratory outcomes [2,3,4,6].

From the point of view of the respiratory pathology of the former premature infants, several groups of patients could be individualized. The first group is one of the premature infants that present with bronchopulmonary dysplasia (BPD) that are discharged from the maternity hospital while still receiving treatment with supplementary oxygen [3]. This is a group in which the clinical picture is clear, and the follow-up protocol is standardized [3]. The second group is represented by the patients with different types of cerebral palsy [7, 8], where the respiratory pathology is especially represented by aspiration pneumonia, apneic spells, and other consequences of neurologic disorders [8].

The third group is represented by the premature infants with or without respiratory disorders in the neonatal period, that present in the first years of life with an increased incidence of respiratory infections [2, 4], or bronchial hyperreactivity [4]. According to a meta-analysis, it is known that the hospital admission rate of the premature infants, especially of those with a gestational age less than 32 weeks and birth weight less than 1500 grams is two times higher than the hospital admission rate of the term neonates (60% versus 20%) [2]. According to the same meta-analysis, the main reason for the hospital admission of the former premature infants is represented by respiratory diseases, namely recurrent wheezing and infection with the respiratory syncytial virus (RSV) [2].

The explanation of the occurrence of particular respiratory conditions in the former premature infants could be that the delivery and the contact with the extrauterine environment take place during a time when the lung is still during its developmental stages that generally occur in utero. When the delivery occurs between 28 and 37 weeks, the lung is in the saccular phase of development [4], and the alveoli are not yet fully developed. Thus, even if the structure of the lung allows the occurrence of the blood gas exchanges, it is not yet completely mature [4], and the interference with the normal developmental process of the lung could make this organ become less efficient in the process of gas exchange and increases the risk for disorders [9]. The functional residual capacity (FRC) of the lung of the former premature neonates is smaller than that of the infants born at term [4], and this difference is maintained throughout the entire childhood until the adult age [10]. Another problem is represented by the deficiency of the airway tethering, which determines the reduction of the radius of the airways during the expiratory phase [4]. It has been demonstrated that the pulmonary expiratory flows are reduced in premature infants during the first year of life and that the smaller the gestational age is, the more pronounced is the abnormality [11]. Also, the expiratory flow is reduced in the case of the former premature infants during the first years of life, and this tendency is persistent until adulthood [10].

As a consequence of these findings, there is an increasing trend in research to identify risk factors for the apparition of this type of pathology in the different categories of premature infants; an example could be The Prematurity Respiratory Outcomes Program (PROP) [3], a research program that by collecting data from the population of premature neonates is aiming to identify the categories at risk for the occurrence of respiratory morbidity [3].

The hypothesis of our research was that there are risk factors among the family history data, gestational age or the pathology in the neonatal period that could identify premature infants at risk for the apparition of the respiratory diseases in this population (respiratory infections that need hospitalization, wheezing, chronic lung disorder (CLD) (also known as bronchopulmonary dysplasia - BPD) and allow the application of prophylactic strategies in this category of infants.

The aim of our study was to determine by statistical correlations the risk factors from the above-mentioned categories that could identify premature infants with gestational age between 30-34 weeks at risk for the occurrence of respiratory pathology during the first two years of life in order to apply preventive and early intervention strategies, similar to the ones used in the premature neonates at risk for neurologic sequelae.

## Material and Methods

### Study Sample

The sample studied was represented by 31 premature neonates with a gestational age between 30-34 weeks , monitored in the program for neonates at risk at Life Memorial Hospital between 2016-2018. The interval for monitoring was at least one year. The study was retrospective and had the approval of the Ethics Committee of Life Memorial Hospital.

### Variables studied

The source of the data was represented by the medical files of the patients and the files of the follow-up program.

The variables studied were represented by antenatal risk factors (familial, maternal or fetal), anthropometric data (gestational age, birth weight), conditions, and treatments in the neonatal period. The antenatal and familial risk factors were represented by the presence of atopy or asthma in the parents, smoking (maternal or in the family), age of the mother. The factors from the neonatal period that were studied were: the presence of respiratory distress syndrome, need for mechanical ventilation, treatment with antibiotics, treatment with reserve antibiotics, presence of CLD/BPD.

There were also noted the following prognostic variables, describing the respiratory pathology of the former premature infants: the presence of infections with RSV and other viral infections requiring hospitalization, the occurrence of wheezing requiring hospitalization and nasal colonization with certain bacteria (*Staphylococcus aureus, Streptococcus*).

### Statistical methods

The data were analyzed using an SPSS 18.0 Software, with a statistical significance of 95% (p< 0.05)

Different tests were applied for the calculation of the significant difference between two or more groups. The tests were chosen according to the distribution of the values of the variables, being considered statistically significant at a limit of 95%. In the case of the quantitative variables, the following were applied:

• The t-Student test – a parametric test used in the case of 2 groups with normal distributions;
• The F-test (ANOVA) used in the case of the comparison of 2 or more mean values from groups with normal distributions. After using the ANOVA test, a Bonferroni correction was applied (post-hoc Bonferroni). This correction reduces the risk of error when testing more than one hypothesis;
• The Chi-Square test was used to compare two or more distributions of two or more frequencies from the same population; it is applied when the expected events are mutually exclusive. When a frequency was small, the Yates correction was applied in order to obtain a more significant estimation of the difference. The Kruskal-Wallis correlation compared ordinal variables from 3 or more groups. The “Pearson” Correlation Coefficient was used when the correlation of two variables from the same group was needed, the sign of the coefficient stating the direct/indirect correlation.

The multiple linear regression aimed to identify a statistically significant relationship between a dependent variable and some independent variables (explaining variables, predictors). By using the software, regression models were built for the appearance of the different types of the above-mentioned respiratory conditions occurring in the former premature infants in the first years of life.

## Results

### Characteristics of the study sample

There were evaluated 31 premature infants. The distribution of the birth-weights and gestational ages are shown in Table 1. The mean birthweight was 1950g, the mean gestational age was 33weeks. There was a homogenous distribution of the values and this allowed the application of the tests for statistical significance (Figure 1 a, b).

**Table 1:** Birthweight and gestational age.

	Mean	Median	Standard Deviation	Variance	Minimum	Maximum	Percentiles
**Birthweight (g)**	1950	2000	371.52	19.05	1100	2890	25 (1840) 50 (2000) 75 (2140)
**Gestational ge (weeks)**	33.03	33	0.95	0.9	31	34	25 (33) 50 (33) 75 (34)

**Figure 1 a:**
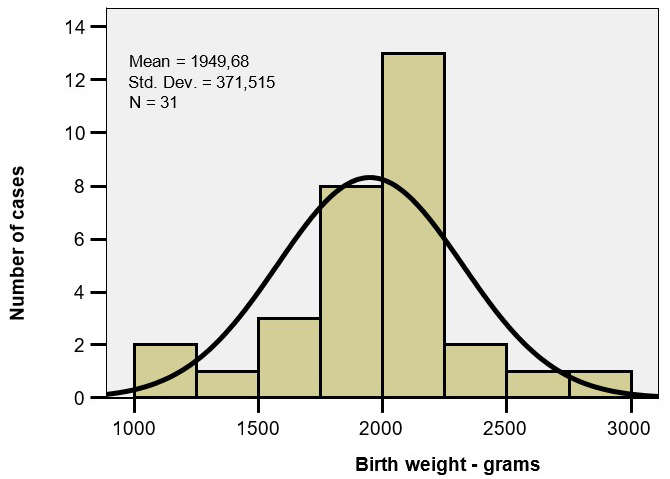
Distribution of birth weights

**Figure 1 b:**
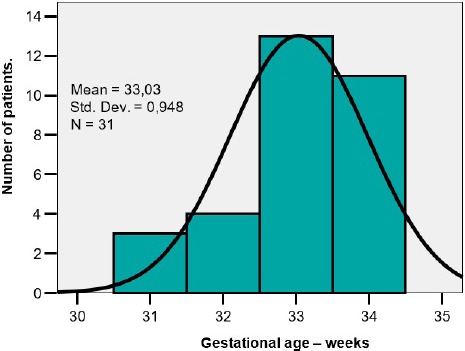
Distribution of gestational ages

The research of the antenatal risk factors showed the following results: the mean age of the mothers at delivery was 30.81years, with a median of 34 years and a standard deviation of + 7.73 years. There was a homogenous distribution, and this allowed the application of tests for statistical significance. Smoking by family members was identified in 11 cases (35.5%); in 4 cases (12.9%), the mother smoked during the pregnancy. Atopy was present in 4 cases in first degree relatives of the former premature infants (mother and father) (12.9%). In 4 of the 31 former premature infants, the family lived in the suburbs, but in the majority of cases (27/31), the family was living in the city.

Regarding the conditions in the neonatal period, the respiratory distress syndrome (RDS) was diagnosed in 20 of the premature infants (64.5%) and the transient tachypnea of the newborn in 7 of the patients (22.6%). Nineteen of the premature infants received respiratory support (9 were ventilated in SIMV (synchronized intermittent mandatory ventilation) mode, and 10 received CPAP (continuous positive airway pressure), the diagnosis of RDS being most frequently associated with the risk of respiratory support (18/20, p<0.001, RR 0.11, CI: 95% 0.03-0.42). The duration of the mechanical ventilation was 2.35 days (1-4 days) with a median of 2 days and a standard deviation of +0.99. The distribution of the values was homogenous, allowing the application of tests for statistical significance. The duration of the oxygen therapy was 3.65 + 2.14 days (0-8 days), the series of values being also homogenous.

The antibiotics were administered in 28/31 premature infants (90.3%). The duration of the treatment varied from 0 to 15 (mean 5.61 + 2.91 days, close to the median value (5 days). Skewness and Kurtosis tests showed that there was a homogenous distribution of the values. There were administered two regimes of antibiotics – the standard regimen (ampicillin + gentamycin in17 patients) and the second line (reserve) of antibiotics (meropenem + gentamycin, meropenem –monotherapy or meropenem + vancomycin in 11 patients).

**Figure 2:**
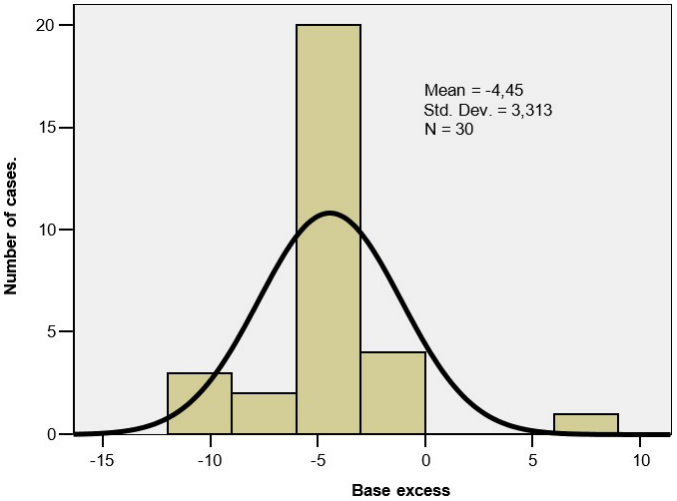
Base excess in the umbilical cord – histogram

The data regarding the base excess from the umbilical cord blood sample can be seen in Table 2. The series of values had a homogenous distribution (Figure 2).

**Table 2:** Base excess in the umbilical.

**Samples**		30
**Mean**		-4.45
**Median**		-4.10
**Standard Deviation**		3.31
**Variance**		10.98
**Skewness Test**		1.175
**Std Error Skewness**		0.427
**Minimum**		-12
**Maximum**		8
**Percentile**	25	-5.05
	20	-410
	75	-3.60

The respiratory conditions after the neonatal period are represented by BPD (2 cases), recurrent wheezing – 7 cases (22.6%), RSV infections requiring hospital admission – 3 patients (9.67%) and other viral infections requiring hospitalization (16 cases). Nasal colonization with *Staphylococcus aureus* was identified in 6 of the former premature infants.

### Research of the correlation between antenatal and neonatal risk factors and the respiratory conditions in the post-neonatal period

Respiratory distress syndrome (RDS) (63.3%) and viral infections (72.7%) were the conditions more frequently found in the premature infants exposed to smoking in the family, but the risk estimated by prematurity was 2.5 times greater in the children with viral infections (RR=2.50; CI: 95%, 0.81-7.70) (Table 3).

**Table 3:** Relation between respiratory infections and the exposure to smoking.

Varable	Exposed to smoking (n=11)		Non-exposed to smoking (n=20)		p	RR	IC95%
	n	%	n	%			
**RDS**	7	63.6	13	65.0	0.619	1.02	0.59-1.77
**Transient tachypnea**	3	27.3	4	20.0	0.484	1.29	0.46-3.59
**CLD**	0	0	2	10.0	0.409	-	-
**Wheezing**	3	27.3	4	20.0	0.484	1.29	0.46-3.59
**RSV**	2	18.2	1	5.0	0.281	2.07	0.79-5.44
**Viral infections**	8	72.7	8	40.0	0.085	2.50	0.81-7.70

Chronic lung disease was identified in 2 premature infants. The multivariate analysis did not identify a significant influence of the prenatal risk factors in the occurrence of CLD (Table 4).

**Table 4:** Linear regression model Dependent variable chronic lung disease. Independent variables – prenatal risk factors.

Model	R	R Square	Adjusted R Square	Std. Error of the Estimate	Change Statistics				
					R Square Change	F Change	df1	df2	Sig. F Change
1	0.101(a)	0.010	-0.024	0.430	0.010	0.298	1	29	0.589
2	0.114(b)	0.013	-0.058	0.437	0.003	0.080	1	28	0.779
3	0.143(c)	0.020	-0.089	0.443	0.007	0.202	1	27	0.657
4	0.149(d)	0.022	-0.128	0.451	0.002	0.0751	1	26	0.824
5	0.150(e)	0.022	-0.173	0.4460	0.000	0.004	1	25	0.947

a Predictors: (Constant), Birth weight (BW)

b Predictors: (Constant), BW, Gestational age (GA)

c Predictors: (Constant), BW, GA, Smoking in the family

d Predictors: (Constant), BW, GA, Smoking in the family, Living in the city/suburbs

e Predictors: (Constant), BW, GA, Smoking in the family, Living in the city/suburbs, age of the mother

The base excess in the umbilical cord blood sample was significantly lower in premature infants with BPD/CLD (- 7.30 vs – 4.25; p=0.044); the difference was not observed in the case of the premature infants with wheezing (-3.31 vs -4.80; p=0.307), RSV infection (-4.07 vs -4.50; p=0.836) or other viral infections(-5.13 vs -3.69; p=0.242) (Figure 3 a, b, c, d)

**Figure 3 a:**
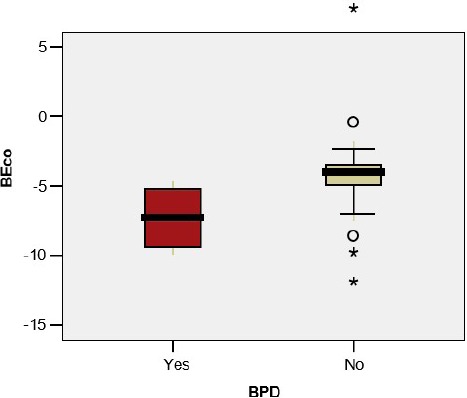
**BEco** - Base excess in the umbilical cord, **BPD** - Bronchopulmonary Dysplasia.

**Figure 3 b:**
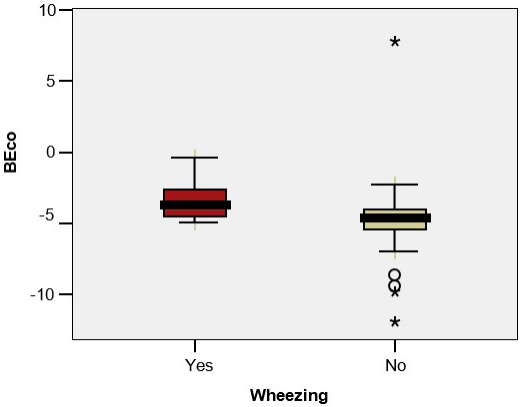
**BEco** - Base excess in the umbilical cord.

**Figure 3 c:**
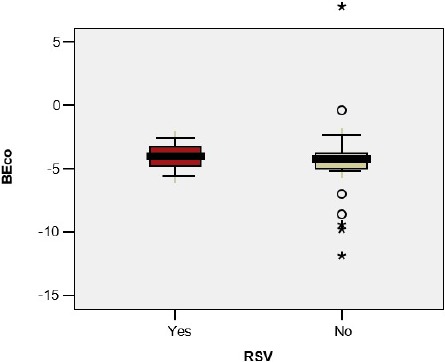
**BEco** - Base excess in the umbilical cord, **RSV** - Respiratory Syncytial Virus.

**Figure 3 d:**
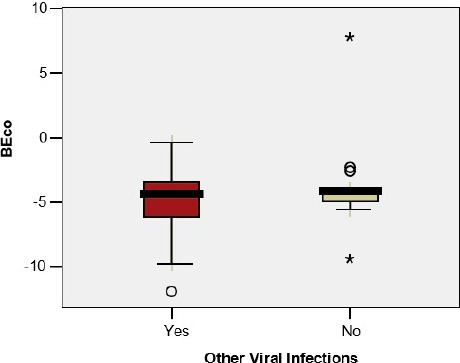
**BEco** - Base excess in the umbilical cord

**Figure 4:**
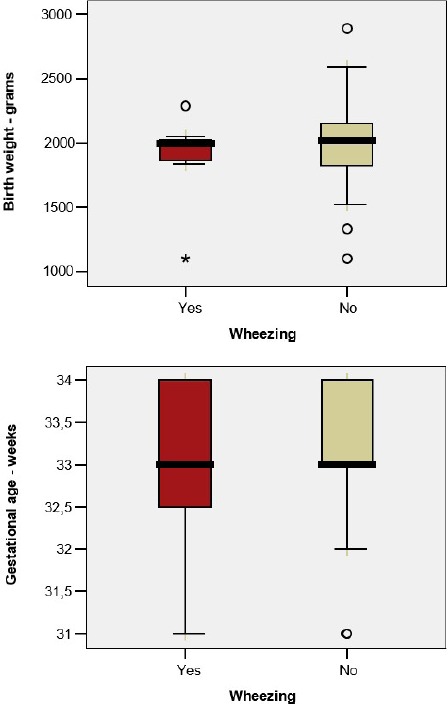
Correlation between the wheezing and the birthweight and the gestational age.

Wheezing was identified in 7 former premature infants (22.6%). In the case of premature infants with wheezing, no association was observed between the occurrence of wheezing and the birth weight (1881 vs. 1970g; p=0.589) or the gestational age (33.0 vs. 33.04 weeks; p=0.921) (Figure 4).

The prenatal risk factors were not good predictors for the occurrence of wheezing (p< 0.05) (Table 5).

**Table 5:** Linear regression model. Dependent variable- wheezing. Independent variables – prenatal risk factors.

Model	R	R Square	Adjusted R Square	Std. Error of the Estimate	Change Statistics				
					R Square Change	F Change	df1	df2	Sig. F Change
1	0.101(a)	0.010	-0.024	0.430	0.010	0.298	1	29	0.589
2	0.114(b)	0.013	-0.058	0.437	0.003	0.080	1	28	0.779
3	0.143(c)	0.020	-0.089	0.443	0.007	0.202	1	27	0.657
4	0.149(d)	0.022	-0.128	0.451	0.002	0.051	1	26	0.824
5	0.150(e)	0.022	-0.173	0.4460	0.000	0.004	1	25	0.947

a Predictors: (Constant), Birth weight (BW)

b Predictors: (Constant), BW, Gestational age (GA)

c Predictors: (Constant), BW, GA, Smoking in the family

d Predictors: (Constant), BW, GA, Smoking in the family, Living in the city/suburbs

e Predictors: (Constant), BW, GA, Smoking in the family, Living in the city/suburbs, age of the mother

The multivariate analysis showed that the gestational age and familial atopy are good predictors for the risk of RSV infections (Table 6). No correlations were demonstrated between the risk of RSV infection requiring hospitalization and smoking in the family or maternal smoking during the pregnancy, intrauterine growth restriction treatment with antibiotics or treatment with reserve antibiotics, duration of the treatment with antibiotics.

**Table 6:** Risk for severe RSV infection. Regression models.

Model		Unstandardized Coefficients		Standardized Coefficients	t	Sig.	95% Confidence Interval for B	
		B	Std. Error	Beta			Lower Bound	Upper Bound
**1**	**(Constant)**	4.234	1.896		2.233	0.033	0.356	8.113
	**GA**	-0.071	0.057	-0.223	-1.230	0.229	-0.188	0.047
**2**	**(Constant)**	4.420	1.934		2.285	0.030	0.458	8.382
	**GA**	-0.070	0.058	-0.221	-1.209	0.237	-0.189	0.049
	**Atopy**	-0.109	0.161	-0.123	-0.673	0.506	-0.439	0.222
**3**	**(Constant)**	3.848	2.083		1.847	0.076	-0.427	8.122
	**GA**	-0.061	0.059	-0.193	-1.028	0.313	-0.183	0.061
	**Atopy**	-0.090	0.164	-0.102	-0.546	0.590	-0.426	0.247
	**Smoking in pregnancy**	0.130	0.167	0.147	0.776	0.445	-0.213	0.473
**4**	**(Constant)**	3.789	2.084		1.818	0.081	-0.494	8.072
	**GA**	-0.062	0.059	-0.197	-1.047	0.305	-0.185	0.060
	**Atopy**	-0.088	0.164	-0.100	-0.538	0.595	-0.426	0.249
	**Smoking in pregnancy**	0.068	0.178	0.077	0.381	0.706	-0.298	0.434
	**Smoking in family**	0.125	0.124	0.198	1.007	0.323	-0.130	0.381
**5**	**(Constant)**	3.643	2.150		1.694	0.103	-0.785	8.071
	**GA**	-0.056	0.062	-0.177	-0.897	0.379	-0.185	0.073
	**Atopy**	-0.057	0.185	-0.065	-0.308	0.760	-0.437	0.323
	**Smoking in pregnancy**	0.059	0.183	0.067	0.323	0.749	-0.317	0.435
	**Smoking in family**	0.124	0.126	0.197	0.984	0.335	-0.136	0.385
	**Intra-uterine Growth Restriction**	-0.058	0.147	-0.086	-0.396	0.696	-0.362	0.245
**6**	**(Constant)**	3.932	2.204		1.784	0.087	-0.617	8.481
	**GA**	-0.073	0.067	-0.231	-1.090	0.287	-0.212	0.065
	**Atopy**	-0.030	0.190	-0.034	-0.156	0.877	-0.422	0.362
	**Smoking in pregnancy**	0.103	0.194	0.117	0.534	0.599	-0.296	0.503
	**Smoking in family**	0.090	0.136	0.142	0.662	0.514	-0.190	0.370
	**Intra-uterine Growth Restriction**	-0.047	0.150	-0.070	-0.317	0.754	-0.356	0.261
	**Antibiotics**	0.166	0.224	0.166	0.743	0.465	-0.296	0.628
**7**	**(Constant)**	3.867	2.247		1.721	0.099	-0.781	8.515
	**GA**	-0.068	0.069	-0.216	-0.988	0.333	-0.211	0.075
	**Atopy**	-0.048	0.198	-0.055	-0.245	0.809	-0.458	0.361
	**Smoking in pregnancy**	0.110	0.197	0.125	0.557	0.583	-0.299	0.518
	**Smoking in family**	0.098	0.139	0.156	0.706	0.487	-0.190	0.387
	**Intra-uterine Growth Restriction**	-0.050	0.152	-0.073	-0.326	0.748	-0.364	0.265
	**Antibiotics**	0.178	0.229	0.178	0.776	0.446	-0.296	0.651
	**Reserve antibiotics**	-0.058	0.130	-0.093	-0.444	0.661	-0.327	0.211
**8**	**(Constant)**	4.234	2.300		1.841	0.079	-0.537	9.005
	**GA**	-0.067	0.070	-0.212	-0.965	0.345	-0.211	0.077
	**Atopy**	0.009	0.210	0.011	0.045	0.965	-0.426	0.445
	**Smoking in pregnancy**	0.123	0.199	0.139	0.616	0.544	-0.290	0.536
	**Smoking in family**	0.096	0.140	0.152	0.684	0.501	-0.195	0.387
	**Intra-uterine Growth Restriction**	-0.087	0.159	-0.128	-0.545	0.591	-0.417	0.243
	**Antibiotics**	0.014	0.299	0.014	0.048	0.962	-0.607	0.635
	**Reserve antibiotics**	-0.123	0.151	-0.199	-0.812	0.425	-0.437	0.191
	**Days on antibiotics**	-0.034	0.040	-0.290	-0.855	0.402	-0.118	0.049

a Dependent Variable: Respiratory Syncytial Virus Infection

The multivariate analysis did not find a model of predictors for respiratory infections, other infections than RSV or bacterial colonization, or recurrent wheezing (Table 7 a,b,c,d).

**Table 7:** Regression models independent variables – other viral infections 7a; all viral infections 7b, bacterial colonisation 7c, recurrent wheezing 7d.

Table 7a								
Model		Unstandardized Coefficients		Standardized Coefficients	t	Sig.	95% Confidence Interval for B	
		B	Std. Error	Beta			Lower Bound	Upper Bound
**1**	**(Constant)**	2.077	3.286		0.632	0.532	-4.644	8.797
	**GA**	-0.018	0.099	-0.033	-0.180	0.858	-0.221	0.185
**2**	**(Constant)**	2.599	3.307		0.786	0.439	-40.176	9.374
	**GA**	-0.016	0.099	-0.031	-0.166	0.869	-0.219	0.186
	**Atopy**	-0.305	0.276	-0.205	-1.106	0.278	-0.870	0.260
**3**	**(Constant)**	0.249	3.368		0.074	0.942	-6.661	7.159
	**GA**	0.020	0.096	0.038	0.209	0.836	-0.177	0.217
	**Atopy**	-0.227	0.265	-0.153	-0.857	0.399	-0.772	0.317
	**Smoking in pregnancy**	0.533	0.270	0.357	1.969	0.059	-0.022	1.088
**4**	**(Constant)**	0.180	3.399		0.053	0.958	-6.807	7.166
	**GA**	0.019	0.097	0.035	0.193	0.848	-0.181	0.218
	**Atopy**	-0.226	0.268	-0.151	-0.843	0.407	-0.776	0.325
	**Smoking in pregnancy**	0.460	0.291	0.308	1.582	0.126	-0.138	1.057
	**Smoking in family**	0.148	0.203	0.138	0.727	0.473	-0.269	0.565
**5**	**(Constant)**	0.073	3.516		0.021	0.984	-7.168	7.315
	**GA**	0.023	0.102	0.043	0.228	0.822	-0.187	0.234
	**Atopy**	-0.203	0.302	-0.136	-0.672	0.508	-0.825	0.419
	**Smoking in pregnancy**	0.453	0.299	0.304	1.518	0.141	-0.162	1.068
	**Smoking in family**	0.147	0.207	0.138	0.711	0.484	-0.279	0.573
	**Intra-uterine Growth Restriction**	-0.042	0.241	-0.037	-0.176	0.862	-0.539	0.454
**6**	**(Constant)**	0.508	3.611		0.141	0.889	-6.945	7.960
	**GA**	-0.002	0.110	-0.005	-0.022	0.982	-0.230	0.225
	**Atopy**	-0.162	0.311	-0.109	-0.520	0.608	-0.804	0.480
	**Smoking in pregnancy**	0.520	0.317	0.349	1.639	0.114	-0.135	1.174
	**Smoking in family**	0.095	0.222	0.089	0.428	0.673	-0.364	0.554
	**Intra-uterine Growth Restriction**	-0.026	0.245	-0.023	-0.106	0.916	-0.531	0.480
	**Antibiotics**	0.250	0.366	0.148	0.682	0.502	-0.507	1.006
**7**	**(Constant)**	0.768	3.601		0.213	0.833	-6.681	8.217
	**GA**	-0.022	0.111	-0.041	-0.197	0.845	-0.251	0.207
	**Atopy**	-0.087	0.317	-0.058	-0.273	0.787	-0.742	0.569
	**Smoking in pregnancy**	0.493	0.317	0.331	1.558	0.133	-0.162	1.148
	**Smoking in family**	0.061	0.224	0.057	0.272	0.788	-0.402	0.523
	**Intra-uterine Growth Restriction**	-0.017	0.244	-0.015	-0.071	0.944	-0.522	0.487
	**Antibiotics**	0.204	0.367	0.120	0.554	0.585	-0.556	0.963
	**Reserve antibiotics**	0.232	0.208	0.222	1.113	0.277	-0.199	0.663
**8**	**(Constant)**	0.656	3.746		0.175	0.863	-7.112	8.424
	**GA**	-0.022	0.113	-0.041	-0.196	0.846	-0.257	0.213
	**Atopy**	-0.104	0.342	-0.070	-0.305	0.763	-0.814	0.605
	**Smoking in pregnancy**	0.489	0.324	0.328	1.508	0.146	-0.183	1.162
	**Smoking in family**	0.062	0.228	0.058	0.270	0.790	-0.412	0.535
	**Intra-uterine Growth Restriction**	-0.006	0.259	-0.005	-0.023	0.982	-0.543	0.531
	**Antibiotics**	0.253	0.487	0.150	0.520	0.608	-0.757	-1.264
	**Reserve antibiotics**	0.252	0.247	0.241	1.021	0.318	-0.260	0.763
	**Days on antibiotics**	0.011	0.066	0.052	0.160	0.874	-0.125	0.146

a Dependent Variable: Other Viral Infections-

**Table 7b:** 

Model		Unstandardized Coefficients		Standardized Coefficients	t	Sig.	95% Confidence Interval for B	
		B	Std. Error	Beta			Lower Bound	Upper Bound
**1**	**(Constant)**	3.158	3.230		0.978	0.336	-3.449	9.765
	**GA**	-0.053	0.098	-0.099	-0.538	0.594	-0.253	0.147
**2**	**(Constant)**	3.805	3.209		1.186	0.246	-2.769	10.379
	**GA**	-0.051	0.096	-0.096	-0.529	0.601	-0.248	0.146
	**Atopy**	-0.378	0.267	-0.257	-1.412	0.169	-0.926	0.170
**3**	**(Constant)**	1.939	3.345		0.580	0.567	-4.924	8.802
	**GA**	-0.022	0.095	-0.041	-0.228	0.821	-0.218	0.174
	**Atopy**	-0.316	0.264	-0.215	-1.200	0.241	-0.857	0.225
	**Smoking in pregnancy**	0.423	0.269	0.287	1.575	0.127	-0.128	0.974
**4**	**(Constant)**	1.831	3.325		0.551	0.587	-5.003	8.666
	**GA**	-0.024	0.095	-0.045	-0.252	0.803	-0.219	0.171
	**Atopy**	-0.314	0.262	-0.213	-1.198	0.242	-0.852	0.225
	**Smoking in pregnancy**	0.309	0.284	0.210	1.088	0.287	-0.275	0.894
	**Smoking in family**	0.230	0.198	0.218	1.158	0.257	-0.178	0.638
**5**	**(Constant)**	1.546	3.426		0.451	0.656	-5.509	8.602
	**GA**	-0.012	0.100	-0.022	-0.117	0.908	-0.217	0.193
	**Atopy**	-0.253	0.294	-0.172	-0.858	0.399	-0.859	0.354
	**Smoking in pregnancy**	0.292	0.291	0.198	1.004	0.325	-0.307	0.891
	**Smoking in family**	0.228	0.201	0.216	1.004	0.268	-0.187	0.643
	**Intra-uterine Growth Restriction**	-0.114	0.235	-0.101	-0.485	0.632	-0.598	0.370
**6**	**(Constant)**	2.045	3.505		0.583	0.565	-5.189	9.278
	**GA**	-0.041	0.107	-0.078	-0.386	0.703	-0.262	0.179
	**Atopy**	-0.205	0.302	-0.140	-0.680	0.503	-0.829	0.418
	**Smoking in pregnancy**	0.368	0.308	0.250	1.196	0.243	-0.267	1.004
	**Smoking in family**	0.169	0.216	0.160	0.782	0.442	-0.277	0.615
	**Intra-uterine Growth Restriction**	-0.095	0.238	-0.084	-0.399	0.693	-0.586	0.396
	**Antibiotics**	0.287	0.356	0.172	0.806	0.428	-0.448	1.021
**7**	**(Constant)**	2.344	3.457		0.678	0.505	-4.808	9.496
	**GA**	-0.063	0.106	-0.120	-0.597	0.557	-0.284	0.157
	**Atopy**	-0.119	0.304	-0.081	-0.391	0.700	-0.749	0.511
	**Smoking in pregnancy**	0.338	0.304	0.229	1.111	0.278	-0.291	0.966
	**Smoking in family**	0.129	0.215	0.123	0.603	0.552	-0.315	0.573
	**Intra-uterine Growth Restriction**	-0.085	0.234	-0.075	-0.363	0.720	-0.569	0.399
	**Antibiotics**	0.233	0.352	0.140	0.662	0.514	-0.496	0.962
	**Reserve antibiotics**	0.266	0.200	0.258	1.331	0.196	-0.147	0.680
**8**	**(Constant)**	2.560	3.590		0.713	0.483	-4.884	10.005
	**GA**	-0.063	0.109	-0.119	-0.578	0.569	-0.288	0.162
	**Atopy**	-0.085	0.328	-0.058	-0.259	0.798	-0.765	0.595
	**Smoking in pregnancy**	0.345	0.311	0.235	1.111	0.279	-0.299	0.990
	**Smoking in family**	0.128	0.219	0.121	0.584	0.565	-0.326	0.582
	**Intra-uterine Growth Restriction**	-0.107	0.248	-0.095	-0.431	0.671	-0.622	0.408
	**Antibiotics**	0.137	0.467	0.082	0.294	0.772	-0.832	1.106
	**Reserve antibiotics**	0.228	0.236	0.221	0.964	0.345	-0.262	0.718
	**Days on antibiotics**	-0.020	0.063	-0.102	-0.323	0.750	-0.151	0.110

a Dependent Variable: All viral infections

**Table 7c:** 

Model		Unstandardized Coefficients		Standardized Coefficients	t	Sig.	95% Confidence Interval for B	
		B	Std. Error	Beta			Lower Bound	Upper Bound
**1**	**(Constant)**	4.019	2.567		1.566	0.128	-1.230	9.268
	**GA**	-0.067	0.078	-0.158	-0.862	0.396	-0.226	0.092
**2**	**(Constant)**	4.396	2.592		1.696	0.101	-0.913	9.704
	**GA**	-0.066	0.078	-0.156	-0.849	0.403	-0.225	0.093
	**Atopy**	-0.220	0.216	-0.186	-1.018	0.318	-0.662	0.223
**3**	**(Constant)**	3.065	2.728		1.124	0.271	-2.532	8.663
	**GA**	-0.045	0.078	-0.107	-0.581	0.566	-0.205	0.115
	**Atopy**	-0.176	0.215	-0.149	-0.818	0.420	-0.617	0.265
	**Smoking in pregnancy**	0.302	0.219	0.256	1.376	0.180	-0.148	0.751
**4**	**(Constant)**	3.028	2.769		1.094	0.284	-2.664	8.720
	**GA**	-0.046	0.079	-0.108	-0.581	0.566	-0.208	0.117
	**Atopy**	-0.175	0.218	-0.149	-0.802	0.430	-0.623	0.273
	**Smoking in pregnancy**	0.263	0.237	0.223	1.109	0.278	-0.224	0.749
	**Smoking in family**	0.079	0.165	0.093	0.477	0.638	-0.261	0.419
**5**	**(Constant)**	2.606	2.824		0.923	0.365	-3.210	8.421
	**GA**	-0.028	0.082	-0.066	-0.339	0.738	-0.197	0.141
	**Atopy**	-0.084	0.243	-0.072	-0.347	0.731	-0.584	0.415
	**Smoking in pregnancy**	0.237	0.240	0.201	0.988	0.333	-0.257	0.731
	**Smoking in family**	0.076	0.166	0.091	0.461	0.649	-0.266	0.419
	**Intra-uterine Growth Restriction**	-0.169	0.194	-0.187	-0.873	0.391	-0.568	0.230
**6**	**(Constant)**	2.284	2.904		0.787	0.439	-3.710	8.278
	**GA**	-0.009	0.088	-0.021	-0.099	0.922	-0.191	0.174
	**Atopy**	-0.115	0.250	-0.097	-0.458	0.651	-0.631	0.402
	**Smoking in pregnancy**	0.187	0.255	0.159	0.735	0.469	-0.339	0.714
	**Smoking in family**	0.115	0.179	0.136	0.642	0.527	-0.254	0.484
	**Intra-uterine Growth Restriction**	-0.181	0.197	-0.201	-0.920	0.367	-0.588	0.225
	**Antibiotics**	-0.185	0.295	-0.139	-0.628	0.536	-0.793	0.423
**7**	**(Constant)**	2.189	2.957		0.740	0.467	-3.928	8.306
	**GA**	-0.002	0.091	-0.004	-0.018	0.986	-0.190	0.187
	**Atopy**	-0.142	0.260	-0.121	-0.547	0.590	-0.681	0.396
	**Smoking in pregnancy**	0.197	0.260	0.167	0.759	0.456	-0.340	0.735
	**Smoking in family**	0.128	0.184	0.151	0.695	0.494	-0.252	0.507
	**Intra-uterine Growth Restriction**	-0.184	0.200	-0.204	-0.921	0.367	-0.599	0.230
	**Antibiotics**	-0.168	0.301	-0.126	-0.558	0.582	-0.792	0.455
	**Reserve antibiotics**	-0.085	0.171	-0.103	-0.496	0.624	-0.439	0.269
**8**	**(Constant)**	2.905	2.967		0.979	0.338	-3.249	9.058
	**GA**	0.001	0.090	0.002	0.007	0.994	-0.185	0.187
	**Atopy**	-0.030	0.271	-0.025	-0.110	0.914	-0.592	0.532
	**Smoking in pregnancy**	0.222	0.257	0.188	0.865	0.397	-0.311	0.755
	**Smoking in family**	0.123	0.181	0.145	0.677	0.505	-0.253	0.498
	**Intra-uterine Growth Restriction**	-0.257	0.205	-0.285	-1.252	0.224	-0.683	0.169
	**Antibiotics**	-0.487	0.386	-0.364	-1.260	0.221	-1.287	0.314
	**Reserve antibiotics**	-0.212	0.195	-0.257	-1.086	0.289	-0.617	0.193
	**Days on antibiotics**	-0.067	0.052	-0.423	-1.291	0.210	-0.175	0.041

a Dependent Variable: Bacterial colonization

**Table 7d:** 

Model		Unstandardized Coefficients		Standardized Coefficients	t	Sig.	95% Confidence Interval for B	
		B	Std. Error	Beta			Lower Bound	Upper Bound
**1**	**(Constant)**	1.498	2.750		0.545	0.590	-4.127	7.123
	**GA**	0.008	0.083	0.019	0.101	0.921	-0.162	0.179
**2**	**(Constant)**	1.942	2.766		0.702	0.488	-3.724	7.608
	**GA**	0.010	0.083	0.021	0.116	0.908	-0.160	0.179
	**Atopy**	-0.260	0.231	-0.208	-10.126	0.270	-0.732	0.213
**3**	**(Constant)**	0.623	2.926		0.213	0.833	-5.380	6.626
	**GA**	0.030	0.084	0.067	0.361	0.721	-0.141	0.202
	**Atopy**	-0.216	0.231	-0.173	-0.937	0.357	-0.689	0.257
	**Smoking in pregnancy**	0.299	0.235	0.240	10.273	0.214	-0.183	0.781
**4**	**(Constant)**	0.608	2.981		0.204	0.840	-5.519	6.735
	**GA**	0.030	0.085	0.067	0.351	0.728	-0.145	0.205
	**Atopy**	-0.216	0.235	-0.173	-0.919	0.367	-0.698	0.267
	**Smoking in pregnancy**	0.283	0.255	0.227	1.111	0.277	-0.241	0.807
	**Smoking in family**	0.032	0.178	0.036	0.179	0.860	-0.334	0.398
**5**	**(Constant)**	0.512	3.084		0.166	0.870	-5.839	6.862
	**GA**	0.034	0.090	0.076	0.379	0.708	-0.151	0.219
	**Atopy**	0.195	0.265	-0.156	-0.736	0.468	-0.741	0.351
	**Smoking in pregnancy**	0.277	0.262	0.222	1.060	0.299	-0.262	0.817
	**Smoking in family**	0.031	0.181	0.035	0.172	0.865	-0.342	0.405
	**Intra-uterine Growth Restriction**	-0.039	0.211	-0.040	-0.182	0.857	-0.474	0.397
**6**	**(Constant)**	0.143	3.169		0.045	0.964	-6.397	6.682
	**GA**	0.056	0.097	0.125	0.579	0.568	-0.143	0.255
	**Atopy**	-0.230	0.273	-0.184	-0.842	0.408	-0.794	0.334
	**Smoking in pregnancy**	0.221	0.278	0.177	0.794	0.435	-0.354	0.795
	**Smoking in family**	0.075	0.195	0.084	0.386	0.703	-0.328	0.478
	**Intra-uterine Growth Restriction**	-0.053	0.215	-0.055	-0.245	0.809	-0.496	0.391
	**Antibiotics**	-0.212	0.322	-0.150	-0.660	0.515	-0.876	0.451
**7**	**(Constant)**	0.143	3.244		0.044	0.965	-6.567	6.853
	**GA**	0.056	0.100	0.125	0.559	0.581	-0.151	0.262
	**Atopy**	-0.230	0.286	-0.184	-0.805	0.429	-0.821	0.361
	**Smoking in pregnancy**	0.221	0.285	0.177	0.774	0.447	-0.369	0.811
	**Smoking in family**	0.075	0.201	0.084	0.374	0.712	-0.341	0.492
	**Intra-uterine Growth Restriction**	-0.053	0.220	-0.055	-0.239	0.813	-0.507	0.402
	**Antibiotics**	-0.212	0.331	-0.150	-0.642	0.527	-0.896	0.472
	**Reserve antibiotics**	0.000	0.188	0.000	0.002	0.998	-0.388	0.389
**8**	**(Constant)**	0.868	3.273		0.265	0.793	-5.920	7.656
	**GA**	0.058	0.099	0.130	0.587	0.563	-0.147	0.263
	**Atopy**	-0.116	0.299	-0.093	-0.387	0.702	-0.736	0.504
	**Smoking in pregnancy**	0.246	0.283	0.197	0.868	0.395	-0.342	0.834
	**Smoking in family**	0.070	0.200	0.079	0.352	0.728	-0.344	0.484
	**Intra-uterine Growth Restriction**	-0.126	0.226	-0.132	-0.557	0.583	-0.595	0.343
	**Antibiotics**	-0.535	0.426	-0.378	-1.256	0.222	-1.418	0.348
	**Reserve antibiotics**	-0.128	0.215	-0.147	-0.596	0.557	-0.575	0.318
	**Days on antibiotics**	-0.068	0.057	-0.405	-1.185	0.249	-0.187	0.051

a Dependent Variable: Recurrent wheezing

## Discussion

The present study is investigating the respiratory prognosis of premature infants between 31-34 weeks. From this point of view, it is probably the first such study in Romanian patients, which is one of the strong points of the research. The weak points of the study are represented by the small number of patients and the absence of a control sample, represented by neonates born at term. A control sample could indeed show the differences regarding the respiratory pathology between preterm and term infants. However, these differences are already known from several studies, and meta-analyses [1-5] and new research with the same result could only multiply the number of studies from this field, obtaining similar results.

The hypothesis of our group was that there is an increased incidence of respiratory diseases in former premature infants and small children compared with the children born at term, and we tried to identify the risk factors for these conditions. Even if the sample is a small one, we aimed to have a homogenous sample concerning the different diagnoses and therapies that could offer the ground to answer the questions of the families regarding the risk factors and the prognosis based on local data, as indicated by the actual guidelines [12].

The choice of studying the 31-34 weeks gestational age range was not random. It is already known that in the case of premature infants of 30 weeks or less, CLD/BPD represents the main type of respiratory disease [3] and the respiratory infections are especially related to RSV; national prevention plans being developed [13] in the case of premature infants delivered at 31-34 weeks gestational age, the respiratory outcome is less clear, even if these premature infants are a bigger population than the neonates born at less than 30 weeks gestational age [3,4]. This is why we considered useful to investigate this category of former preemies in order to obtain risk factors and prognosis data for it as well.

Concerning the characteristics of the studied sample, it could be affirmed that they are specific to this population of premature infants: the occurrence of the respiratory distress syndrome in more than half of the patients (20/31), the need for respiratory support (19/31) and a slight predominance of CPAP ventilation over SIMV (10/9) [14]. The duration of the ventilation is also a short one, specific to the mild or medium RDS – 1-4 days, with a median of 2 days (48 hours) [15]. Also, regarding the treatment with antibiotics, the duration was short (mean 5.61, median 5 days); the antibiotic regimen was, most of the time, a standard one (ampicillin + gentamycin).

The incidence of chronic lung disease (CLD/ BPD) in the sample was 2/31 patients. Even if the incidence is rather small, it is interesting the occurrence of this type of disease in premature infants with a gestational age greater than 30 weeks. A reason for this could be the current definition of CLD that includes oxygen dependency at more than 36 weeks postmenstrual age [16]. From the point of view of the risk factors, the only identified risk was represented by an increased base excess in the umbilical artery sample at delivery. We could speculate that the pathogenic cascade leading to CLD begins in the moment of delivery, and the prevention of the birth asphyxia and strategies of gentle ventilation in this group of neonates could prevent the disease.

Regarding the antenatal risk factors, the study showed an association between smoking by family members and the risk of appearance of the respiratory distress syndrome (RDS) and viral infections. The increased risk of RDS related to smoking is somehow surprising, considering the data from the literature, which suggests that a smoking mother or family member represents a protective factor against the occurrence of RDS [17]. New data from the medical literature modulate this statement. First of all, we must consider that the population studied is represented by premature infants from well-cared pregnancies, in which steroid prophylaxis was administered. It is proven that smoking diminishes the effect of the corticosteroids on the maturation of the fetal lung, the incidence of RDS being greater in the neonates of smoking mothers receiving corticosteroids than in the neonates of non-smoking mothers receiving the same treatment [18]. Other effects of maternal smoking on the lung and the respiratory system of the fetus and neonate could be clustered as the fetal tobacco syndrome [19], which is an intoxication of the fetus with different substances from the cigarettes (nicotine and others) that affect the alveoli and airways of the fetus [19]. Three effects were identified: the decrease in the number of alveoli with the decrease in the number of secondary bronchi and ramifications of the bronchial tree, the decrease in the respiratory volume, and the premature aging” of the fetal lung [19]. Regarding the post-neonatal period, it is proven that smoking in the family increases the risk of respiratory tract infections in children during the first years of life [20,21], also identified in our research. A smoking mother or family member was not identified as a risk factor for recurrent wheezing in the former premature infant, as opposed to the data from the medical literature [20,22,23]. One explanation could be that wheezing is a multifactorial disease; also, certain studies indicate that this factor acts mostly associated with other factors, especially in the case of patients with a low socioeconomic class [20,22,23], but this was not the case in the current study. 

In the multivariate regression model of the risk of RSV infections requiring hospitalization, low gestational age, and atopy in the family were identified as independent risk factors. There are studies indicating that the risk of a severe RSV infection is determined by genetic factors that diminish the immune answer to RSV, factors related to maternal conditions (atopy, asthmatic mothers or family members), factors related to the ante- or postnatal environment (smoking mother, family history of smoking, pollution), conditions in the neonatal period (prematurity, artificial feeding, chronic lung disorder, cardiac malformations, mechanical ventilation) and others [24]. Low gestational age was found to be an important risk factor for the severity of RDS [25,26,27]. Even if the incidence of severe RSV infections is smaller than that seen in the medical literature in the same category of patients (10% versus 25%), the explanation, as for several of the above-mentioned risk factors, was that the patients come from families with a medium-high socioeconomic status, that implies conditions of life with a minimal risk of infections and an increased protection of the children by hygiene measures. Atopy is also a risk factor mentioned in other studies [28, 29]. The explanation of the role of atopy as a risk factor for the occurrence of severe RSV infections is most probably related to the genetic factors involved, and an immune deficiency that could determine both types of pathology [24]. 

Almost one-quarter of premature infants in the studied sample presented with wheezing episodes requiring hospital admission during the first two years of life. The number is bigger when compared to the one found in the infants born at term. Neither the gestational age nor other ante- or postnatal factors were identified as being statistically significant associated with the occurrence of wheezing in the regression model. This fact could be interpreted as proof of the fact that the risk of occurrence of recurrent wheezing is constant throughout this sub-population of premature infants. In an editorial from 2013, it is written that this category of premature neonates should be regarded as seriously as possible concerning the respiratory diseases in the post-neonatal period, especially those affecting the airways (wheezing, asthma) [30]. In addition, there are studies that demonstrate that the expiratory volume is already decreased, and the respiratory resistance is already increased at 40 weeks corrected age in this category of premature infants [4,31].

## Conclusions

Premature delivery occurs in a moment when the entire body of the fetus is still developing. Previous research focused mainly on the consequences of premature delivery and pathology of premature infants on the development of the central nervous system. Our study shows, for the first time in Romania, that in the case of preterm neonates, there are long-term respiratory consequences: chronic lung disease, an increased incidence of severe RSV infections, recurrent wheezing, and viral infections. The research is even more important as it concerns a group of premature infants considered to be at low risk – those with a gestational age of 30-34 weeks.

We believe that based on the results of the many studies in this field, including our research, a component regarding the early diagnosis and prevention of the respiratory pathology should be included in the follow up programs for premature infants. In this way, risk factors and respiratory disorders in former premature infants could be identified and treated earlier; also, complex strategies for prevention could be elaborated, starting from the antenatal and perinatal period.

## Conflict of Interest

The authors confirm that there are no conflicts of interest.
